# High-quality-draft genome sequence of the heavy metal resistant and exopolysaccharides producing bacterium *Mucilaginibacter pedocola* TBZ30^T^

**DOI:** 10.1186/s40793-018-0337-8

**Published:** 2018-11-28

**Authors:** Xia Fan, Jingwei Tang, Li Nie, Jing Huang, Gejiao Wang

**Affiliations:** State Key Laboratory of Agricultural Microbiology, College of Life Science and Technology, Huazhong Agricultural University, Wuhan, 430070 People’s Republic of China

**Keywords:** *Mucilaginibacter pedocola*, Genome sequence, Heavy metal resistance, Exopolysaccharides

## Abstract

*Mucilaginibacter pedocola* TBZ30^T^ (= CCTCC AB 2015301^T^ = KCTC 42833^T^) is a Gram- negative, rod-shaped, non-motile and non-spore-forming bacterium isolated from a heavy metal contaminated paddy field. It shows resistance to multiple heavy metals and can adsorb/remove Zn^2+^ and Cd^2+^ during cultivation. In addition, strain TBZ30^T^ produces exopolysaccharides (EPS). These features make it a great potential to bioremediate heavy metal contamination and biotechnical application. Here we describe the genome sequence and annotation of strain TBZ30^T^. The genome size is 7,035,113 bp, contains 3132 protein-coding genes (2736 with predicted functions), 50 tRNA encoding genes and 14 rRNA encoding genes. Putative heavy metal resistant genes and EPS associated genes are found in the genome.

## Introduction

The genus *Mucilaginibacter* was first established by Pankratov et al. in 2007 and the type species is *Mucilaginibacter paludis* [1]. The common characteristics of this genus are Gram-negative, non-spore-forming, non-motile, rod-shaped and producing exopolysaccharides (EPS) [1, 2]. EPS are long-chain polysaccharides and consist of branched, repeating units of sugars or sugar derivatives [3]. EPS producing bacteria play an important role in environmental bioremediation such as water treatment, sludge dewatering and metal removal [4]. So far, genomic features of *Mucilaginibacter* strains are less studied.

*Mucilaginibacter pedocola* TBZ30^T^ (= CCTCC AB 2015301^T^ = KCTC 42833^T^) was isolated from a heavy metal contaminated paddy field in Hunan Province, P. R. China [5]. Here we show that strain TBZ30^T^ is resistant to multiple heavy metals and remove Zn^2+^ and Cd^2+^. In addition, strain TBZ30^T^ is able to produce EPS. The genomic information of strain TBZ30^T^ are provided.

### Organism information

#### Classification and features

**Fig. 1:**
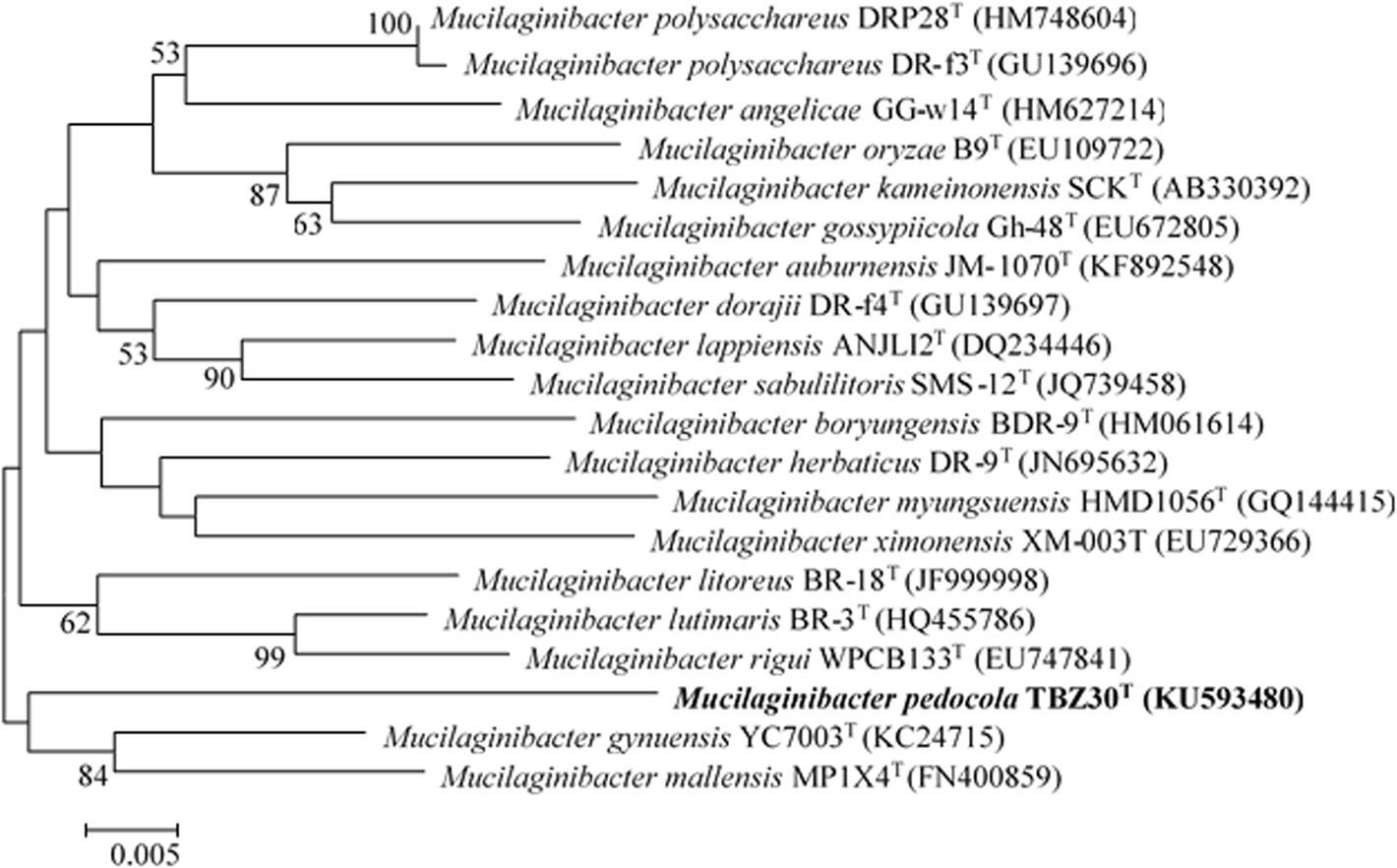
A neighbor-joining phylogenetic tree based on 16S rRNA gene sequences showing the phylogenetic relationships of strain TBZ30^T^ and the related species. The bootstrap value less than 50% are not shown. Bar, 0.005 substitutions per nucleotide position

Similarity analysis was performed using neighbor-joining method based on the 16S rRNA gene sequences and a phylogenetic tree was constructed using MEGA version 6.0 software (Fig. 1). Bootstrap analysis with 1000 replications was conducted to obtain confidence levels of the branches. Strain TBZ30^T^ showed the highest 16S rRNA gene sequence similarity with *Mucilaginibacter gynuensis* YC7003^T^ (95.8%), *Mucilaginibacter mallensis* MP1X4^T^ (95.4%) and *Mucilaginibacter litoreus* BR-18^T^ (95.4%) [6–8] and grouped together with *M. gynuensis* YC7003^T^ (95.8%) and *M. mallensis* MP1X4^T^ (Fig. 1).

**Fig. 2:**
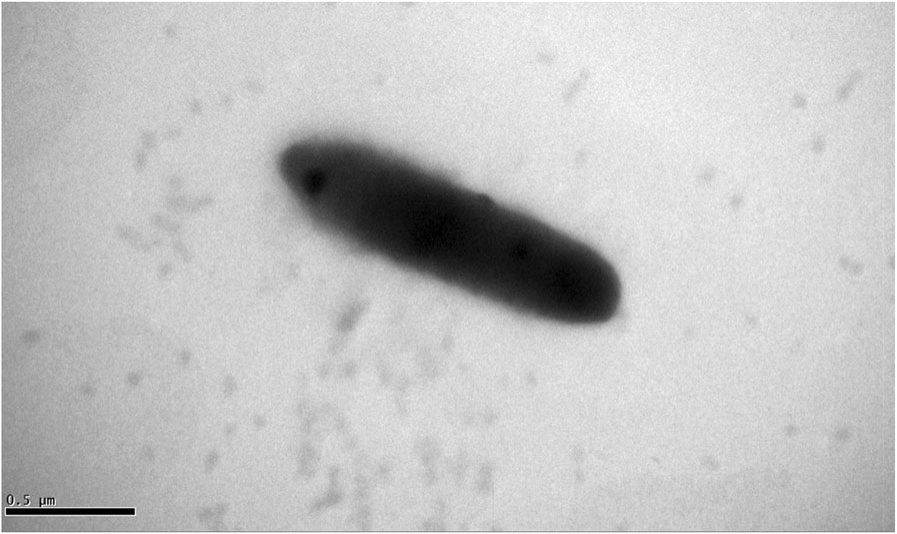
A scanning electron microscope (SEM) image of Mucilaginibacter pedocola TBZ30^T^ cells. The bar scale represents 0.5 μm

**Fig. 3:**
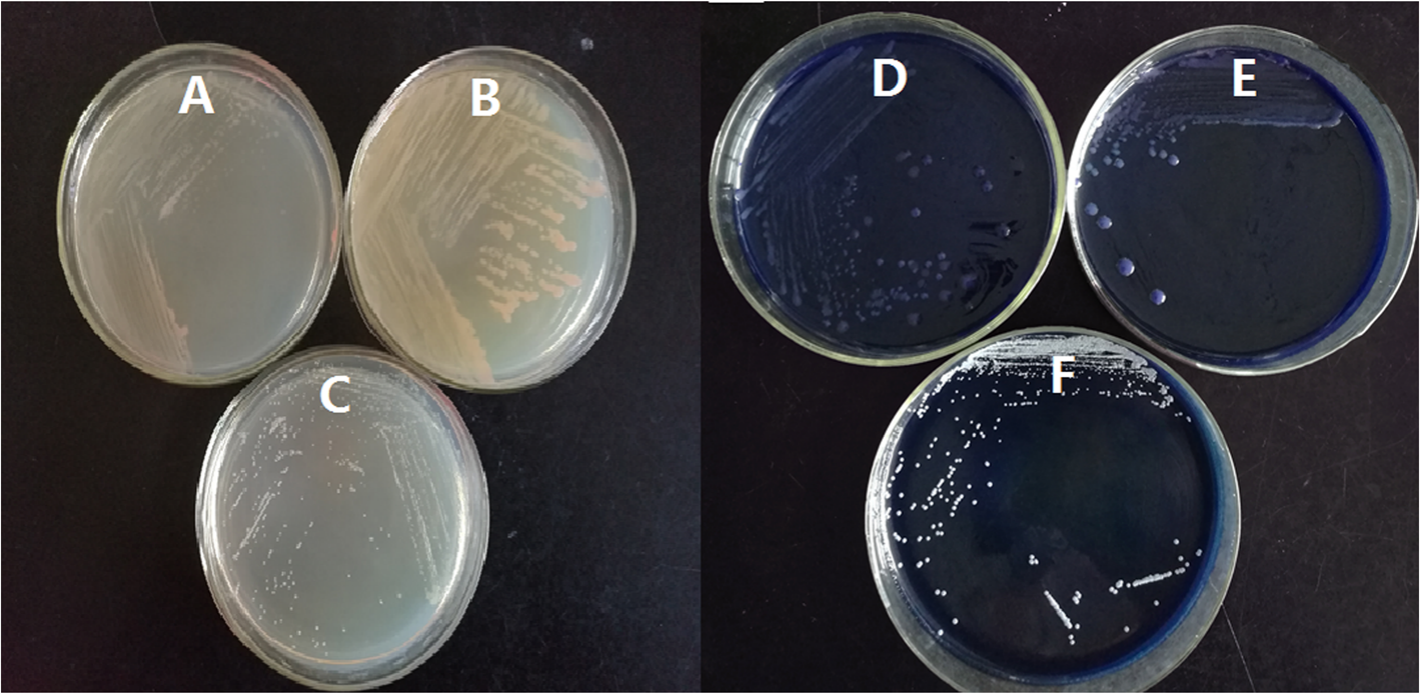
EPS detection using the aniline blue staining method [9]. **a**, **b** and **c** strain TBZ30^T^, positive control Mucilaginibacter litoreus BR-18^T^ and negative control *Nocardioides albus* KCTC 9186^T^ cultivated in LB plates, respectively; (**d**, **e** and **f**) the above three strains cultivated in LB-aniline blue plates, respectively

**Fig. 4:**
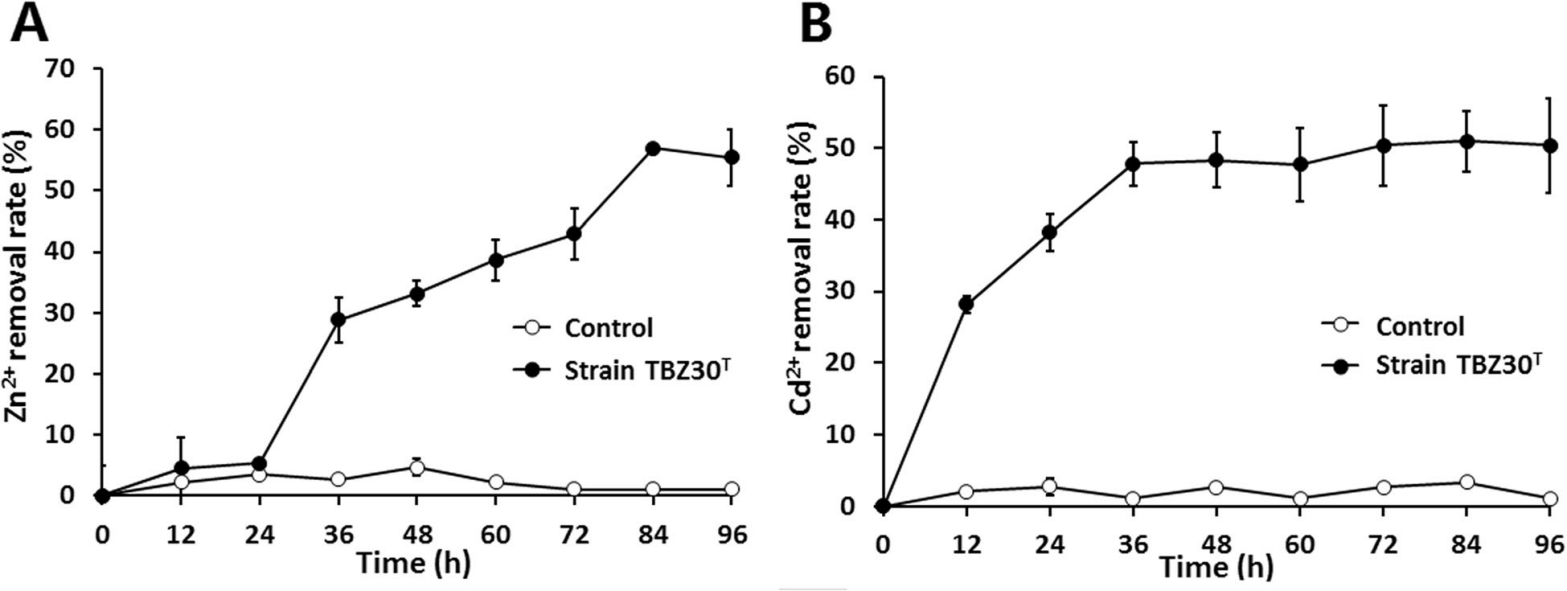
Zn^2+^ and Cd^2+^ removal by strain TBZ30^T^ in R2A liquid media. **a** Zn^2+^ removal by strain TBZ30^T^; (**b**) Cd^2+^ removal by strain TBZ30^T^. The control represents R2A liquid medium with 0.3 mM Zn^2+^ or 0.25 mM Cd^2+^ without the inoculation of strain TBZ30^T^. Data are shown as the mean of three replicates

Strain TBZ30^T^ is Gram-negative, non-motile, and non-spore-forming. Cells are rod-shaped (0.3–0.4 × 1.1–1.3 μm) (Fig. 2). Colonies are circular, pink, convex and smooth on R2A agar. Growth occurs aerobically at 4–28 °C (optimum, 25 °C), pH 5.0–8.5 (optimum, pH 7.0), and in the presence of 0–1.0 (*w*/*v*) NaCl (optimum, without NaCl) (Table 1) [5]. Oxidase- and catalase-positive [5]. It can use glucose, mannose, L-arabinose, maltose, melibiose, rhamnose and glycogen as the sole carbon sources [5]. Strain TBZ30^T^ can produce EPS testing by aniline blue staining method [9] (Fig. 3). The colonies of strain TBZ30^T^ and the known EPS producing strain *M. litoreus* BR-18^T^ are pink on LB plates (Fig. 3a and b), while the colonies are blue on LB-aniline blue plate (Fig. 3d and e). However, the colonies are always white for the negative control *Nocardioides albus* KCTC 9186^T^ [10, 11] on either LB or LB-aniline blue plates (Fig. 3c and f). All of the above strains were incubated at 28 °C for 7 days. In addition, strain TBZ30^T^ is resistant to multiple heavy metals. The minimal inhibition concentration (MIC) tests for different heavy metals were performed on R2A agar plates at 28 °C for 7 days. The MICs for ZnSO_4_, CdCl_2_, PbSO_4_, CuSO_4_ and NaAsO_2_ are 3.5 mM, 1.5 mM, 0.4 mM, 1.2 mM and 0.35 mM, respectively. Furthermore, strain TBZ30^T^ could adsorb/remove nearly 60% of Zn^2+^ and 55% of Cd^2+^ in the R2A liquid medium (added with 0.3 mM ZnSO_4_ and 0.25 mM CdCl_2_, respectively) (Fig. 4). The amount of the heavy metals were detected by an atomic absorption spectrometer.

**Table 1:** Classification and general features of *Mucilaginibacter pedocola* TBZ30^T^ [39]

MIGS ID	Property	Term	Evidence code^a^
	Classification	Domain *Bacteria*	TAS [40]
		Phylum *Actinobacteria*	TAS [41, 42]
		Class *Sphingobacteria*	TAS [43, 44]
		Order *Sphingobacteriales*	TAS [45, 46]
		Family *Sphingobacteriaceae*	TAS [47]
		Genus *Mucilaginibacter*	TAS [1]
		Species *pedocola*	TAS [5]
		Strain TBZ30^T^ (= CCTCC AB 2015301^T^ = KCTC 42833^T^)	
	Gram stain	negative	TAS [5]
	Cell shape	rod	TAS [5]
	Motility	non	TAS [5]
	Sporulation	non-sporulating	NAS
	Temperature range	4–28 °C	TAS [5]
	Optimum temperature	25 °C	TAS [5]
	pH range; Optimum	5.0–8.5, 7.0	TAS [5]
	Carbon source	glucose, mannose, L-arabinose, maltose, melibiose, rhamnose, rhamnose and glycogen	TAS [5]
MIGS-6	Habitat	paddy field with heavy metal	TAS [5]
MIGS-6.3	Salinity	0–1% NaCl (*w*/*v*), optimal at 0%	TAS [5]
MIGS-22	Oxygen requirement	aerobic	TAS [5]
MIGS-15	Biotic relationship	free-living	TAS [5]
MIGS-14	Pathogenicity	non-pathogen	NAS
MIGS-4	Geographic location	Linxiang city, Hunan province, China	TAS [5]
MIGS-5	Sample collection	2014	TAS [5]
MIGS-4.1	Latitude	N30°17′54”	TAS [5]
MIGS-4.2	Longitude	E109°28′16”	TAS [5]
MIGS-4.4	Altitude	not reported	

^a^Evidence code-TAS: Traceable Author Statement (i.e., a direct report exists in the literature); NAS: Non-traceable Author Statement (i.e., not directly observed for the living, isolated sample, but based on a generally accepted property for the species, or anecdotal evidence) [48]

### Genome information

#### Genome project history

*M. pedocola* TBZ30^T^ was sequenced on the basis of its abilities of heavy metals resistance and removal, which has a great potential for bioremediation. The draft genome was sequenced by Wuhan Bio-Broad Co., Ltd., Wuhan, China. The high-quality-draft genome sequence has been deposited at DDBJ/EMBL/GenBank under the accession number MBTF00000000.1. The project information is shown in Table 2.

**Table 2:** Project information

MIGS ID	Property	Term
MIGS-31	Finishing quality	High-quality draft
MIGS-28	Libraries used	Illumina Paired-End library (300 bp insert size)
MIGS-29	Sequencing platforms	Illumina Miseq 2000
MIGS-31.2	Fold coverage	377.50×
MIGS-30	Assemblers	SOAPdenovo v2.04
MIGS-32	Gene calling method	GeneMarkS^+^
	Locus TAG	BC343
	Genbank ID	MBTF00000000.1
	Genbank Date of Release	04, 25, 2017
	GOLD ID	Gs0134261
	Bioproject	PRJNA331061
MIGS-13	Source material identifier	Strain CCTCC AB 2015301
	Project relevance	Bioremediation

#### Growth condition and DNA isolation

*M. pedocola* TBZ30^T^ was grown in R2A medium at 28 °C for 36 h with continuous shaking at 120 rpm. Bacterial cells were harvested through centrifugation (13,400×g for 5 min at 4 °C) and the total genomic DNA was extracted using the QiAamp kit (Qiagen, Germany). The quality and quantity of the DNA were determined using a spectrophotometer (NanoDrop 2000, Thermo).

#### Genome sequencing and assembly

Whole-genome DNA sequencing was performed in Bio-broad Co., Ltd., Wuhan, China using Illumina standard shotgun library and Hiseq2000 pair-end sequencing strategy [12]. For accuracy of assembly, low quality of the original sequence data reads were removed. The assembly of TBZ30^T^ genome is based on 16,967,512 quality reads totaling 2,523,391,653 bases with a 377.50× average genome coverage. The final reads were assembled into 39 contigs (> 200 bp) using SOAPdenovo v2.04 [13]. The part gaps of assembly were filled and the error bases were revised using GapCloser v1.12 [14].

#### Genome annotation

The genome of strain TBZ30^T^ was annotated through the NCBI PGAP, which combined the gene caller GeneMarkS^+^ with the similarity-based gene detection approach [15]. Pseudo genes were predicted using the NCBI PGAP. Internal gene clustering was performed by the OrthoMCL program using Match cutoff of 50% and E-value Exponent cutoff of 1-e5 [16, 17]. The COGs functional categories were assigned by the WebMGA server with E-value cutoff of 1-e10 [18]. The translations of the predicted CDSs were used to search against the Pfam protein family database and the KEGG database [19, 20]. The transmembrane helices and signal peptides were predicted by TMHMM v. 2.0 and SignalP 4.1, respectively [21, 22].

### Genome properties

**Fig. 5:**
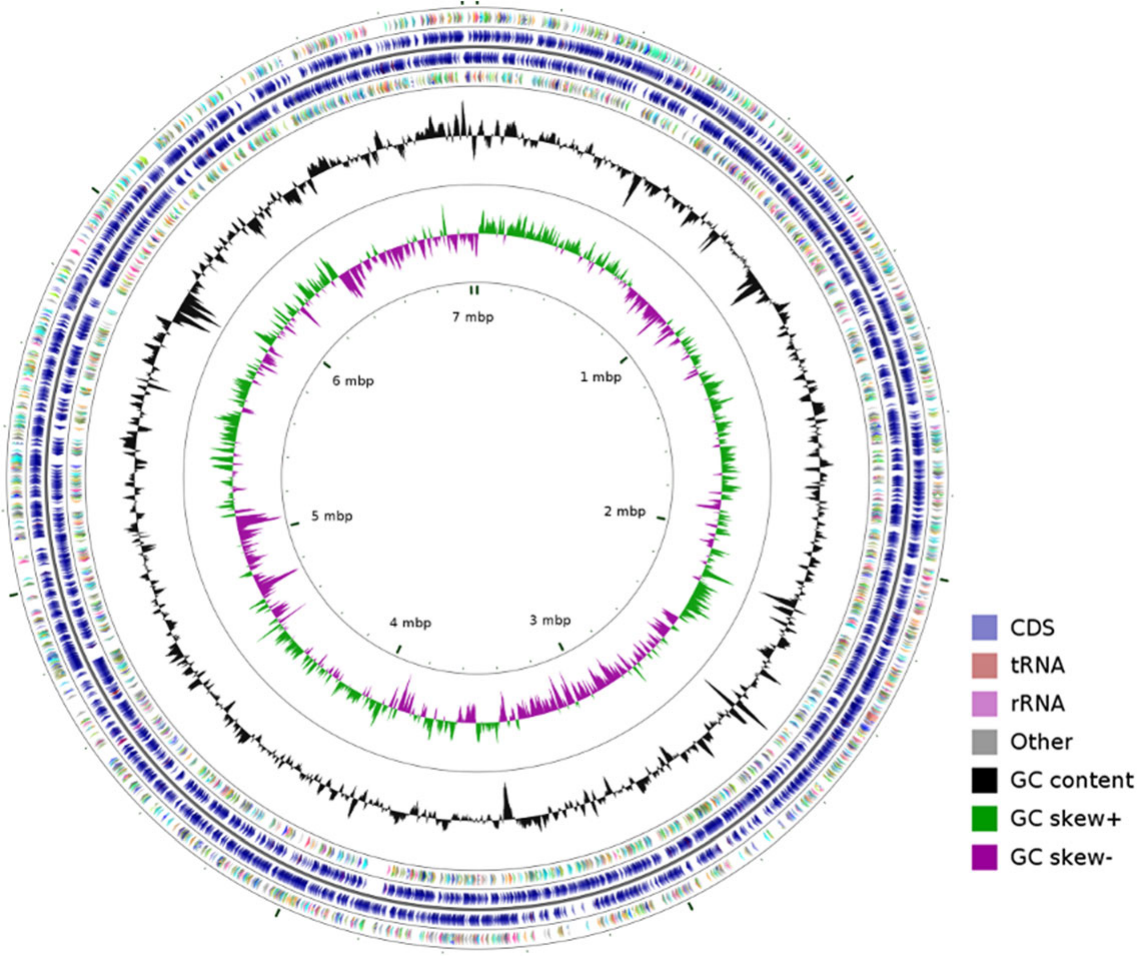
A graphical circular map of Mucilaginibacter pedocola TBZ30^T^. From outside to center, rings 1, 4 show protein-coding genes colored by COG categories on forward/reverse strand; rings 2, 3 denote genes on forward/reverse strand; rings 5 show G + C % content; ring 6 shows G + C % content plot and the innermost ring shows GC skew

The genome size of strain TBZ30^T^ is 7,035,113 bp with an average G + C content of 46.1% (Table 3). It has 6072 genes including 5935 protein-coding genes, 70 pseudo genes and 14 rRNA, 50 tRNA, and 3 ncRNA genes. The information of the genome statistics is shown in Table 3 and the classification of genes into COGs functional categories is summarized in Table 4. The graphical genome map is provided in Fig. 5.

**Table 3:** Nucleotide content and gene count levels of the genome

Attribute	Value	% of total
Genome size (bp)	7,035,113	100
DNA coding (bp)	6,126,065	87.1
DNA G + C (bp)	46.1%	100
DNA scaffolds	38	100
Total genes	6072	100
Protein-coding genes	5935	97.7
RNA genes	67	1.1
Pseudo genes	70	1.2
Genes in internal clusters	587	9.7
Genes with function prediction	2736	45.1
Genes assigned to COGs	4046	66.6
Genes with Pfam domains	4434	73.0
Genes with signal peptides	1005	16.6
Genes with transmembrane helices	1407	23.2
CRISPR repeats	11	0.2

The total is based on the size of the genome in base pairs and the total number of protein coding genes in the annotated genome

**Table 4:** Number of genes associated with the 21 general COG functional categories

COG class	count	% of total	description
J	160	2.70	Translation, ribosomal structure and biogenesis
A	1	0.02	RNA processing and modification
K	406	6.84	Transcription
L	224	3.77	Replication, recombination and repair
B	1	0.02	Chromatin structure and dynamics
D	35	0.59	Cell cycle control, cell division, chromosome partitioning
V	88	1.48	Defense mechanisms
T	459	7.73	Signal transduction mechanisms
M	389	6.55	Cell wall/membrane/envelope biogenesis
N	23	0.39	Cell motility
U	87	1.47	Intracellular trafficking, secretion, and vesicular transport
O	123	2.07	Posttranslational modification, protein turnover, chaperones
C	185	3.12	Energy production and conversion
G	337	5.68	Carbohydrate transport and metabolism
E	247	4.16	Amino acid transport and metabolism
F	73	1.23	Nucleotide transport and metabolism
H	156	2.63	Coenzyme transport and metabolism
I	162	2.73	Lipid transport and metabolism
P	200	3.37	Inorganic ion transport and metabolism
Q	106	1.79	Secondary metabolites biosynthesis, transport and catabolism
R	593	9.99	General function prediction only
S	431	7.26	Function unknown
–	1449	24.41	Not in COGs

The total is based on the total number of protein coding genes in the genome

### Insights from the genome sequence

Strain TBZ30^T^ could be resistant to multiple heavy metals (Zn^2+^, Cd^2+^, Pb^2+^, Cu^2+^ and As^3+^) and adsorb/remove Zn^2+^ and Cd^2+^ during cultivation. Analyzing of its genome, various putative proteins related to multiple heavy metals resistance are found (Table 5). RND efflux systems (CzcABC), CDF efflux systems (CzcD and YieF) and P-type ATPases (HMA and ZntA) are responsible for the efflux of Zn^2+^, Cd^2+^ and Pb^2+^ [23–27]. Zip family metal transporter and P-type ATPase ZosA are associated with the efflux of Zn^2+^, Cd^2+^ or Cu^2+^ [28–30], and CutC is involved in Cu^2+^ homeostasis [30–32]. Moreover, As^3+^ resistant proteins including arsenite efflux pump ACR3, arsenate reductase ArsC, arsenite S-adenosylmethyltransferase ArsM and arsenic resistance repressor ArsR are also found [33–35] (Table 5).

**Table 5:** Putative protein involved in heavy metals resistance and EPS production

Heavy metals or EPS production	Putative function	Locus_tag of the predicted protein
Zinc-Cadmium-Lead resistance		
RND efflux systems	CusA/CzcA heavy metal efflux RND transporter	BC343_14685, BC343_14785
	Efflux RND transporter periplasmic adaptor subunit CzcB	BC343_14680, BC343_14795
	Outer membrane protein CzcC	BC343_14800
CDF efflux systems	Cation transporter CzcD	BC343_11185
	Cation transporter FieF	BC343_27530
P-type ATPase	Heavy metal translocating P-type ATPase HMA	BC343_08790
	Heavy metal translocating P-type ATPase ZosA	BC343_14675
	Cadmium-translocating P-type ATPase ZntA	BC343_00930
Zip super family	Zip family metal transporter	BC343_14670
Copper resistance	Zip family metal transporter	BC343_14670
	Heavy metal translocating P-type ATPase ZosA	BC343_14675
	Copper homeostasis protein CutC	BC343_23340
Arsenic resistance	Arsenite efflux pump ACR3	BC343_02735
	Arsenate reductase ArsC	BC343_02740, BC343_24635
	Arsenite S-adenosylmethyltransferase ArsM	BC343_24640
	Arsenical resistance repressor ArsR	BC343_24645, BC343_02755
Nucleotide sugars biosynthesis for EPS production		
CDP-Glc	Sugar kinase	BC343_21040, BC343_04390
	Phosphoglucomutas	BC343_18360
	Gucose-1-phosphate cytidylyltransferase RfbF	BC343_04660
ADP-Glc	Glucose-1-phosphate adenylyltransferase	BC343_23820
GDP-D-man	Glucose-6-phosphate isomerase	BC343_14065
	6-phosphofructokinase	BC343_20710, BC343_25175
	Mannose-6-phosphate isomerase ManA	BC343_15810, BC343_21400
	Phosphoglucosamine mutase phosphomannomutase	BC343_21600
	Mannose-1-phosphate guanylyltransferase	BC343_03170
EPS biosynthesis	3-Deoxy-D-manno-octulosonic-acid transferase KdtA	BC343_09425
	Priming glycosyltransferase CpsE	BC343_04560
	Glycosyltransferase	BC343_04600, BC343_09445
	ABC transporter KpsMT	BC343_09400, BC343_09585
	Polysaccharide co-polymerase protein PCP	BC343_04670
	Outer membrane polysaccharide protein OPX	BC343_04675
	Flippase Wzx	BC343_08105
	Capsular biosynthesis protein PHP	BC343_09405

Strain TBZ30^T^ produces EPS during cultivation. According to KEGG analysis, the complete biosynthesis pathway of repeating units of nucleotide sugars are identified in the genome, including the biosynthesis of CDP-Glc, ADP-Glc and GDP-D-man (Table 5). Genes related to long-chain polysaccharide assembly are also found (Table 5). The EPS production pathway in strain TBZ30^T^ appears to belong to ABC transporter dependent pathway [36]. First, the 3-deoxy-D-manno-octulosonic-acid transferase (KdtA) is responsible for the synthesis of poly-Kdo linker using either diacyl or monoacyl phosphatidylglycerol as the substrate [36]; Then priming glycosyltransferase (CpsE) catalyzes the transformation of the first repeating unit to the poly-Kdo linker; Next, glycosyltransferases catalyze the synthesis of EPS repeat-unit; Finally, the polymerized repeat-units are exported through an envelope-spanning complex consisting of ABC transporter (KpsMT), polysaccharide co-polymerase protein (PCP) and outer membrane polysaccharide protein (OPX) [37, 38]. In addition, strain TBZ30^T^ genome owns a flippase (Wzx) which catalyzes the translocation of repeat-units crossing the cytoplasmic membrane. EPS have been reported to play an important role in metal removal [3]. Therefore, it is possible that the EPS of strain TBZ30^T^ participate in Zn^2+^ and Cd^2+^ removal by adsorption.

## Conclusions

To the best of our knowledge, this study presents the first genomic information of a *Mucilaginibacter* type strain*.* The data reveal good correlation between genotypes and phenotypes. The genome information and the features provide insights for further theoretical and applied analysis of *M. pedocola* TBZ30^T^ and the related *Mucilaginibacter* members.

## Abbreviations

EPS: Exopolysaccharides
MIC: Minimal inhibition concentration
